# A novel single-feed hybrid reconfigurable microstrip patch antenna for 5G mobile communication and radio frequency energy harvesting applications at 28/38GHz

**DOI:** 10.1371/journal.pone.0260407

**Published:** 2022-01-18

**Authors:** Muhammad Kamran Shereen, Muhammad Irfan Khattak, Farid Zubir, Abdul Basit

**Affiliations:** 1 Electrical Engineering Department, Microwave and Antenna Research Group, University of Engineering and Technology Peshawar, Peshawar, Pakistan; 2 United States Pakistan Centre for Advanced Studies in Energy (USPCASE), University of Engineering and Technology Peshawar, Peshawar, Pakistan; 3 School of Electrical Engineering, Faculty of Engineering, University Teknologi Malaysia, Johor Bahru 81310, Malaysia; Universiti Teknologi Petronas, MALAYSIA

## Abstract

Reconfigurable antennas have received much attention in RF energy harvesting models owing to their selectivity for operating frequency and polarization. The characteristic of having frequency selectivity and polarization selectivity can be termed as frequency diversity and polarization diversity, respectively. This paper investigates a rectenna device with a new proposed topology in order to eliminate coupling between input and output lines and increase the rectification efficiency with the use of single feed hybrid reconfigurable antenna, switch between 28GHz and 38GHz frequency. Moreover, it is designed to charge a rechargeable battery of 1watt(W). The Reconfiguration mechanism is realized by electronically controlling different states of Switches. PIN Diode (as RLC Equivalent circuit) is used as a switch for ON/OFF states. This antenna mainly comprises rectangular shaped patches (28GHz and 38GHz) with Triango-Truncated edge at the corners. Eighteen PIN Diodes are placed symmetrically throughout the antenna presenting as, S1 & S2 for frequency reconfiguration, S3 to S6 & S7 to S10 connects Triango Truncated edge at the corners for polarization reconfiguration, and for radiation pattern reconfiguration at S11 to S14 & S15 to S18 has been used. The proposed antenna model is capable of simultaneously changing, the radiation patterns as clock and anti-clockwise directions at ±90-degree shift in E and H planes, circularly polarized (CP) states among, Linear Polarization (LP), Right Hand Circularly Polarization (RHCP), and Left Hand Circularly Polarization (LHCP). The current design describes using single antenna for energy harvesting and 5G mobile communication application. This would lead to higher output currents, leading to the ability to efficiently charge a wide variety of batteries. A fully functional prototype has been designed, fabricated and its compound reconfiguration characteristics have been validated for simulated and measured results. For validation of results, the experimental results and the simulation results from the proposed mathematical model were made into comparison, and excellent correlation between the measured and simulated results was obtained.

## Introduction

The demand for wirelessly powering sensor nodes has increased exponentially with the increase in number of devices deployed for Internet of Things (IoT). It is not practical to power each device through network of electrical wires especially when the number of deployed nodes is large. Wireless power transfer (WPT) serves as a potential candidate to energize wireless sensor nodes. WPT not only allows freedom of movement but also eliminates the size and weight limitation due to use of batteries and reduces the maintenance cost associated with the finite lifetime of batteries. Due to variations in power level of RF signal in environment and high nonlinearity of rectifier circuit, its dependence on a single band is not practical. Multiband rectifiers are more suitable to provide power by capturing energy from a number of bands simultaneously. Combining single-band rectifiers for multiband energy harvesting increases the size, thus a single-cell multiband rectifier is preferable but its design is a challenge due to complex input impedance.

Various studies have reported single-band millimeter-wave rectifiers operating at 24GHz [1]. Rectifiers operating at 35GHz were also reported in [2]. Ladan and Wu [2] presented harmonic harvester rectifier at 35GHz where power in harmonics was converted to dc to increase the efficiency. Most of the work at millimeter-wave has been done only for single band at either 24 or 35GHz. Riaz et al. [3] presented a single-cell dual-band rectifier for energy-harvesting applications at millimeter-wave frequencies of 28 and 38GHz. We have included millimeter-wave industrial, scientific and medical (ISM) band at 24GHz in addition to millimeter-wave 5G bands of 28 and 38GHz to capture energy from three bands simultaneously which increases the overall dc conversion efficiency [3]. This band is widely used for WPT applications at millimeter-wave frequencies and vehicular radars. The proposed rectifier matched at 24, 28, and 38GHz can be used for both energy-harvesting applications at 5G communication bands (28 and 38GHz) and WPT applications at ISM band (24GHz), simultaneously.

For frequency reconfiguration [4, 5], switching, reactive loading, and tuning materials are generally used. By attaching or removing a certain component (conducting patch) changes the physical length of an antenna increases or decreases the resonant spectrum from its desired frequency to a lower or higher one [6, 7]. An antenna’s polarization reconfigurability depends on the current distribution and direction of flow on its surface [8, 9]. Reconfigurable radiators [10], slot antennas [11], reconfigurable feeding networks [12, 13], parasitic surfaces / meta-surface / partially reflecting surfaces [14, 15] are some of the techniques employed. In the literature, there are several classical beam-steering approaches available: rotating dipole arms or rotating the antenna itself in the orthogonal plane, etc [16]. Phase-controlled array [17], parasitic-patch or stub-controlled antenna [18, 19], pixel-based structure [20, 21], multilayer or stacked configuration [22], CPW-to-slot-line transition [23, 24], monopole or monocone radiator surrounded by a switchable reflector / director array [25], continued research on the nature of beam-steering antennas in various ways. Hybrid/Compound reconfiguration allows multiple antenna parameters, such as frequency and radiation pattern [26] or frequency and polarization [27], to be tuned simultaneously.

Besides communicational applications of 5G, Reconfigurable antennas have received much attention in RF energy harvesting (rectenna) models owing to their selectivity for operating frequency, radiation pattern, and polarization [28]. A rectenna is a particular type of antenna that consists of four main components: antenna, precertification filter, rectifying circuit, and direct-current (DC) pass filter that rectifies incoming electromagnetic waves into DC [29]. From the last decades, the growth of wireless power transmission and satellite solar power transmission rectennas has gained prominence in implementing unique features and applications, such as radio frequency identification systems, sensing batteries or capacitors, wireless local area networks, WiMAX, cognitive radio systems, and in medical applications [30] to increase conversion efficiency. For enhancing the conversion efficiency, various broadband antennas [30], massive antenna arrays [30], and circularly polarized antennas have been designed [30]. Hybrid reconfigurable patch antennas are growing in popularity for their use in wireless systems among different types of microstrip patch antennas. They are used in rectennas due to their low profile, small size, low manufacturing cost, conforming to planer and nonplanar shapes, cheap and ease to fabricate using advanced printed-circuit technologies in terms of choosing specific patch mode for functioning of resonant frequency, polarization, pattern and impedance. Therefore, as integrated antennas in mobile wireless devices and portable devices, they are perfectly recommended to be used.

**Fig 1:**
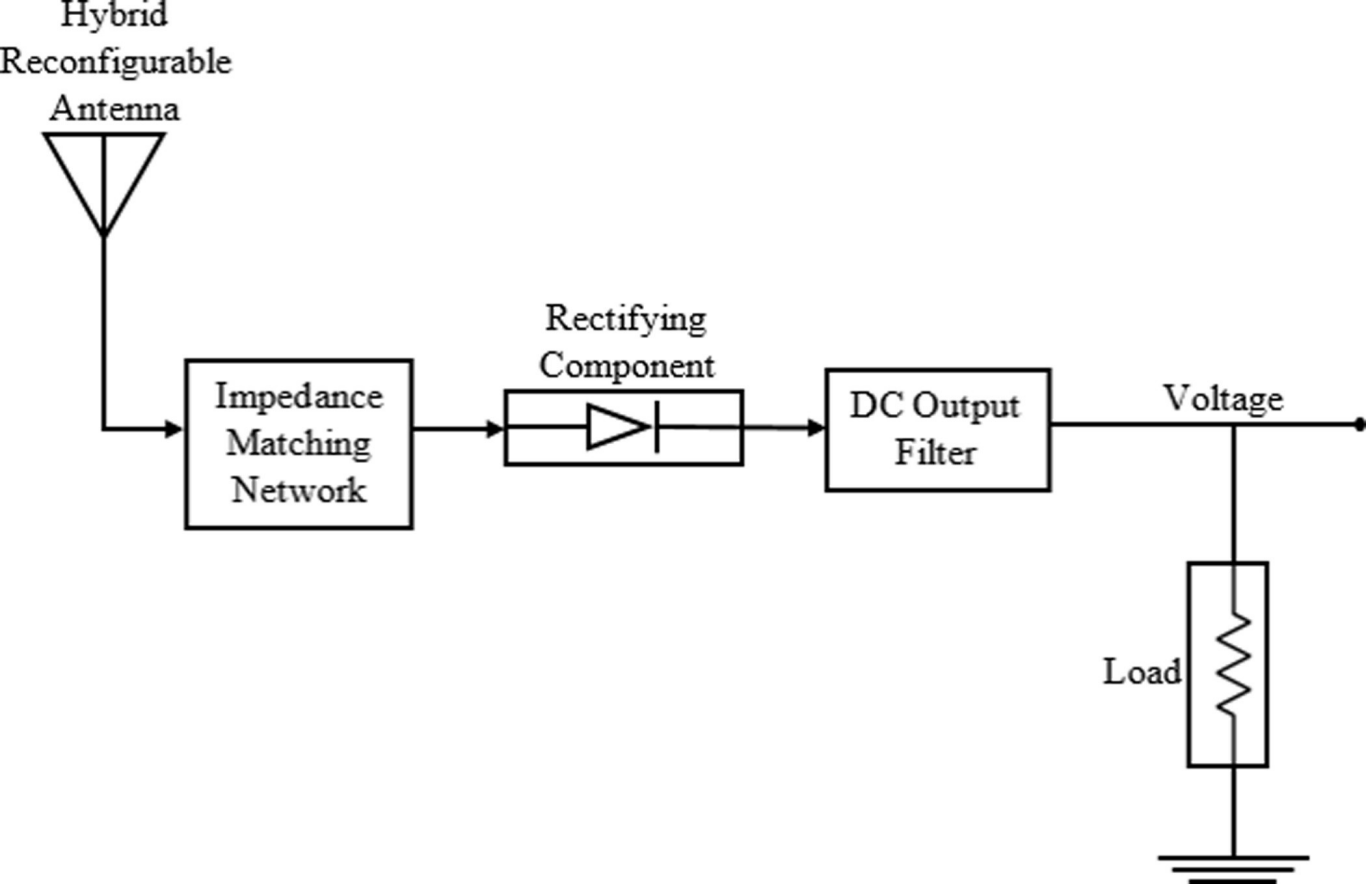
General block diagram of a hybrid reconfigurable rectenna.

A rectifier consists of impedance matching network for transferring maximum power from antenna to rectifier, a diode for rectification of AC signal, an output filter to suppress harmonics generated by diode and finally a load where power is dissipated. A general block diagram of rectenna is shown in Fig 1.

This work contributes to the novelty of designing a single feed antenna for reconfiguration mechanisms such as frequency, pattern, and polarization. All three reconfiguration mechanisms are tuned and controlled independently using switches. Millimeter waves higher frequencies are targeted here, i.e., 28GHz and 38GHz (as per Federal Communications Commission (FCC, Washington, DC, USA) standards/guidelines for 5G mobile communication technology never claimed before [30]. The proposed mechanism is also deployed for RF energy harvesting, which is helpful for researchers who are interested in improving rectenna design to obtain better RF energy harvesting performance, especially in case of obtaining reduced size and reconfigurability in frequency pattern and polarization harmonics rejection never used before. The antenna was designed and tested, and the performance results are summarized in Table 1. An excellent correlation between the measured and mathematical modelled (simulation) results was obtained.

**Table 1:** Performance of the proposed mathematical model antennas.

Active Frequency Patch (GHz)	Gain (dBi)		Bandwidth (GHz)		Return Loss (dB)		VSWR	
	Sim.	Mea.	Sim.	Mea.	Sim.	Mea.	Sim.	Mea.
**P1_28**	9.1	9.01	3.312	3.291	-38.4	-34.5	0.50	0.59
**P2_38**	8.9	8.87	2.359	2.351	-41.8	-37.3	0.53	0.61

## Materials and methods

### Design geometry of proposed hybrid reconfigurable antenna

**Fig 2:**
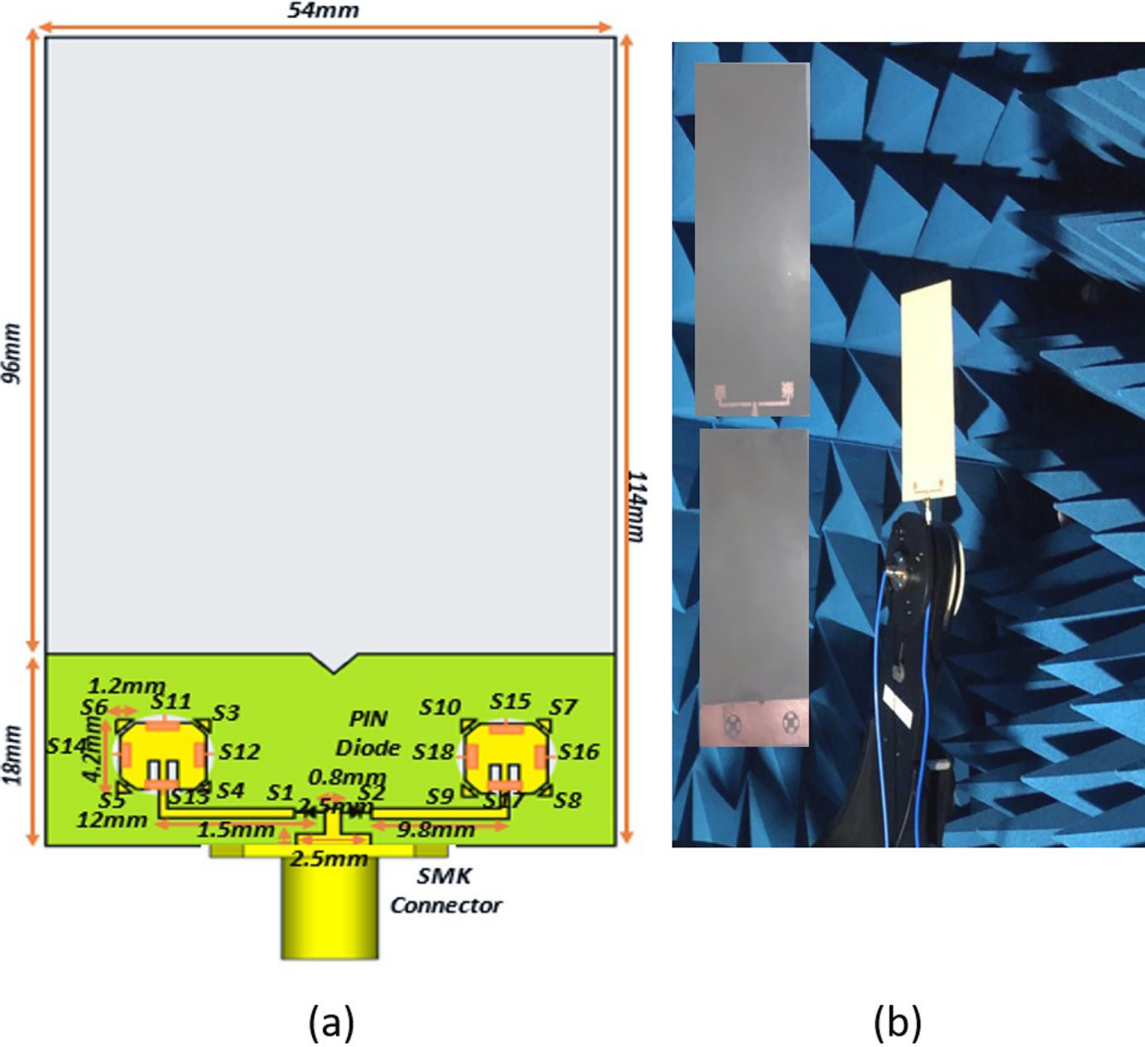
Hybrid reconfigurable antenna designed geometry. (a) Proposed Hybrid Model. (b) fabricated prototype.

In this study we presented hybrid reconfigurable proposed antenna design (the mathematical formulations of the hybrid reconfigurable architecture [28]), as shown in Fig 2A and its fabricated prototype in Fig 2B, which reconfigures frequency, pattern, and polarization by changing the switch position ON/OFF. The notations used in the model are presented in Table 2. Two square patches of side P1_28 and P2_38 is printed on RT Duroid 5880 substrate (εr = 2.2) with thickness *hs* = 0.509mm. A λ/2 impedance transformer connects a 50ohm feed line to the patch for impedance matching. Four (a total of eight from S3-S6 & S7-S10) PIN diodes (switches) are symmetrically placed on each patch to bridge the gap between the center patch and outer Triango-Truncated edge at the corners for polarization reconfiguration. Moreover, four (a total of eight from S11-S14 & S15-S18) parasitic rectangular conductors are formed on the partially grounded conductor for Radiation pattern reconfiguration.

**Table 2:** Nomenclature of important parameters.

Name	Description	Name	Description
S(1–18)	PIN Diodes as Switches from 1 to 18	*opf*	Operating Frequency
P1_28	Patch 1 for 28GHz	*psl*	Patch Side Length
P2_38	Patch 2 for 38GHz	*fd*	Different Frequency

Both the patches P1_28 and P2_38 are connected via two PIN Diodes for frequency reconfiguration. Feedline is controlled with SMK connector. PIN diode used in the proposed antenna is SPDT-PE7170 PIN Diode, and is modeled with the manufacturer’s specified diode equivalent values in the simulation. According to the datasheet [29], ON state of the PIN diode has 3.2MΩ resistance and OFF state has 1.2pF capacitance. The behavior of PIN diode shows as very effective Radio Frequency switch because of its intrinsic properties, which offers additional charge storage in between the two junctions. In switch ON position, it works as a series combination of R/ON and L/ON for conduction (forward-biased/closed circuit) while it works as parallel combination of R/OFF and C/OFF with L/OFF in switch OFF position (reversed biased/open circuit).

**Fig 3:**
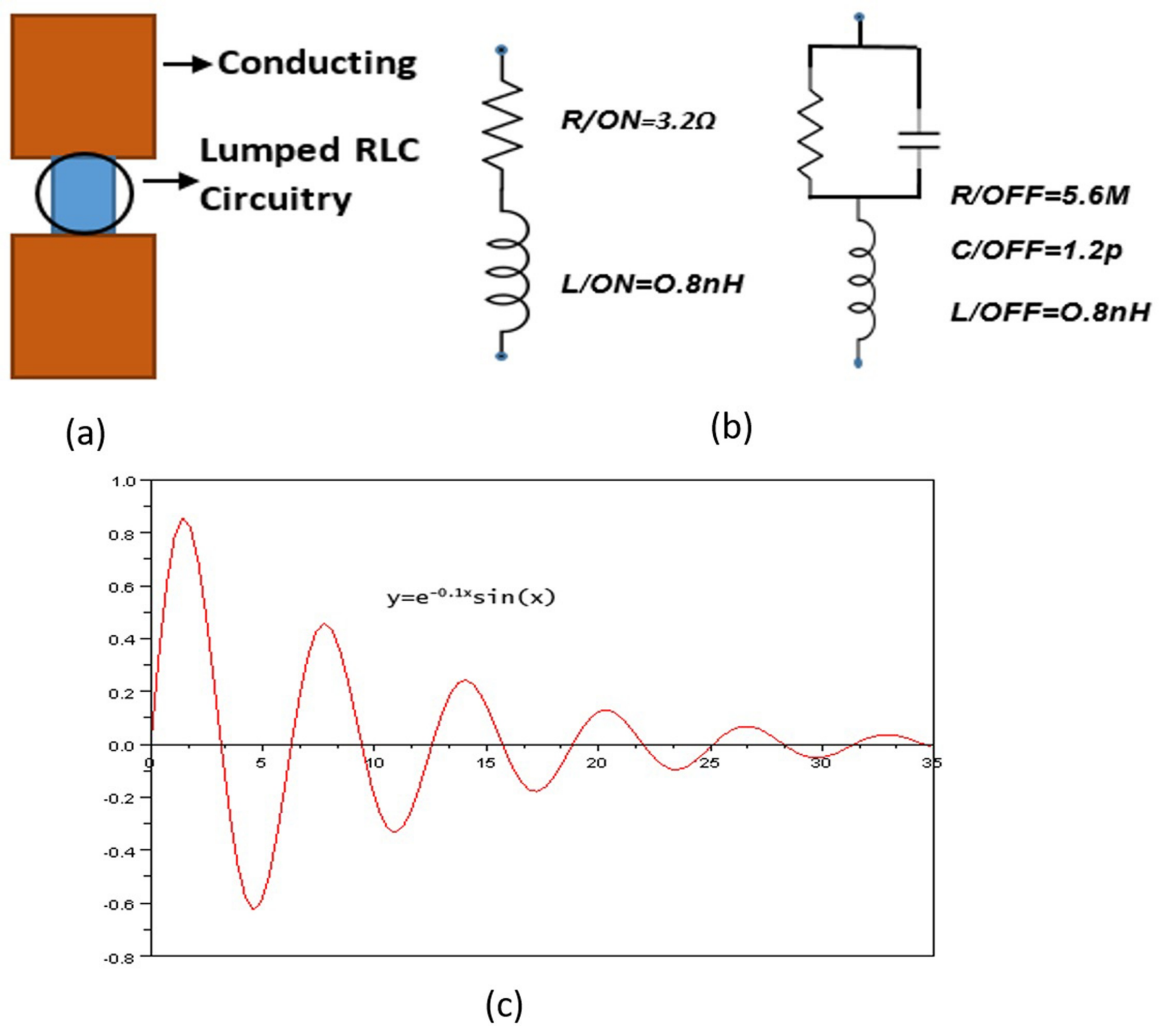
PIN diode structure (a) HFSS PIN Diode Symbol. (b) Ideal equivalent circuit models of PIN diode working at 28/38GHz ON state, OFF state (c) Step Response of RLC switch for oscillation.

Two PIN diode switches were initially modeled for frequency reconfiguration through a lumped element network, with a switch S1 in the ’ON’ configuration, and S2 in the ’OFF’ configuration, as discussed above. With a grid density optimization method of 12 lines per wavelength, the HFSS frequency-domain solver has been used. The configured dimensions maintain adequate resonance matching. As shown in Fig 3, PIN diodes are used to achieve switching positions, i.e. OFF state and ON state. In modeling and simulation of a reconfigurable antenna, RLC equivalent circuit model of the switch is very significant. The HFSS framework of a switch is formed in switch contacts as electrical equivalent circuit using lumped RLC parameters (Fig 3A and 3B).

***For ON state***:

*V*, the voltage source powering the circuit.
*I*, the current admitted through the circuit.
*R*, the effective resistance of the combined load, source, and components.
*L*, the inductance of the inductor component.

When resistor and inductor for ON state is connected with a source to form a circuit (Eqs 1–5), the two components are all in series (when Capacitance is negligible) with the voltage source. The governing differential equation can be found by substituting into Kirchhoff’s voltage law (KVL) the constitutive equation for each of the two elements. From the KVL,

$$V_{R}+V_{L}=V(t)$$ (1)

Where *V*_*R*_ and *V*_*L*_ are the voltages across *R* and *L* respectively and *V*(*t*) is the time-varying voltage from the source. Substituting their values,

$$RI(t)+L\frac{dI(t)}{dt}=V(t),$$ (2)

$$\frac{d^{2}}{dt^{2}}I(t)+\frac{R}{L}\frac{d}{dt}I(t)=0,$$ (3)

Taking the time derivative and dividing by *L* leads to the following second order differential equation

$$\frac{d^{2}}{dt^{2}}I(t)+2\alpha \frac{d}{dt}I(t)=0,(\alpha \;in\;units\;of\;frequency,\;\alpha =\frac{R}{2L})$$ (4)

In the case of the series RL circuit, the damping factor is given by

$$\varsigma =\frac{R}{2\sqrt{L}},$$ (5)

***For OFF state***:

The resonant frequency in this case is given by (Eqs 6–8),

$$\omega_{0}=\sqrt{\frac{1}{LC}-\frac{1}{{\left(RC\right)}^{2}}}$$ (6)

The underdamped response for a hybrid RLC is,

$$I(t)=Be^{-\alpha t}\;\cos(\omega_{d}t)+Be^{-\alpha t}\;\sin(\omega_{d}t)$$ (7)

$$I(t)=Be^{-\alpha t}\;\sin(\omega_{d}t+\varphi )$$ (8)

Where, $\omega_{d}=\sqrt{\omega_{0}^{2}-\alpha^{2}}=\omega_{0}\sqrt{1-\varsigma^{2}}$

This is called the damped resonance frequency. The resonance frequency, *ω*_0_, which is the frequency at which the circuit will resonate when driven by an external oscillation, may often be referred to as the underdamped (Fig 3C) resonance frequency to distinguish it. By placing the value R/ON = 3.2ohm, L/ON = 0.8nH, R/OFF = 5.6M, C/OFF = 1.2p and L/OFF = 0.8nH, the proposed switch RLC equivalent is underdamped.

### Hybrid reconfigurable optimization

This represents the main stage of this work, and it presents the strategy used in developing the set of mathematical function that could be used to evaluate a suitable value for substrate height (*h*_*s*_ *)* for a given antenna design specification. In this work, *h*_*s*_ is chose to be defined using the following simplified mathematical expressions in Eq (9):

$$h_{s}=\left(\frac{\alpha F_{r}\sqrt{\varepsilon_{r}+1}+\beta \sqrt{2C_{o}}}{\sqrt{2C_{o}}+\gamma F_{r}\sqrt{\varepsilon_{r}+1}}\right)$$ (9)

where the *αα*, *ββ*, and *γγ* can be found using an optimization technique. However, the particle swarm optimization (PSO) techniques was chosen. The objective function of the optimization was to minimize the difference between the estimation and true value of the hs. After the optimization, it was found that the model was inaccurate when *αα* and *ββ* are fixed at constant values, however, *γγ* can be fixed at 0.5053. In order to make *αα* and *ββ* vary with change in parameter specification, let *αα* and *ββ* be as shown in Eq (10), respectively:

$$\alpha =X_{1}\left(\begin{array}{l} X_{2}{\left(W_{p}\right)}^{X_{3}}-X_{4}{\left(F_{r}\right)}^{X_{5}}-X_{6}{\left(F_{r}\right)}^{X_{7}}\\ +X_{8}{\left(E_{r}\right)}^{X_{9}}-X_{10}{\left(E_{r}\right)}^{X_{11}}+X_{12} \end{array}\right),$$

$$\beta =Y_{1}\left(\begin{array}{l} Y_{2}{\left(W_{p}\right)}^{X_{3}}-Y_{4}{\left(F_{r}\right)}^{X_{5}}-Y_{6}{\left(F_{r}\right)}^{X_{7}}\\ +Y_{8}{\left(E_{r}\right)}^{X_{9}}-Y_{10}{\left(E_{r}\right)}^{X_{11}}+Y_{12} \end{array}\right)$$ (10)

The set of parameters *X* and *Y* are unknown variables that can be determined using PSO. Where, *C*_*o*_ is the speed of light in air (mm/s); *F*_*r*_ is the desired center frequency (GHz) and and it can be evaluated using the mathematical formulation. Table 3 shows the optimized dimensional parameters of the proposed mathematical modelled antenna at 28GHz. The same procedure was adopted for 38GHz. These differences arise from the manufacturing sensitivity and the effect of the transmissions line feed connector.

**Table 3:** Simulated and measured variation of antenna design parameters with substrate thickness at 28GHz.

*hs* (mm)	*Lp* (mm)	*Wp* (mm)	*Frequency* (GHz)		*Directivity* (dB)		*Gain* (dBi)		*Return Loss* (dB)		*BW* (GHz)	
			Sim.	Mea.	Sim.	Mea.	Sim.	Mea.	Sim.	Mea.	Sim.	Mea.
0.42	18	54	28.453	28.124	9.534	9.111	9.59	9.21	19.343	19.213	1.5435	1.489
0.52	18	54	28.563	28.236	9.433	9.231	9.48	9.11	42.623	42.342	2.1234	2.2442
0.62	18	54	27.692	27.374	9.345	9.276	9.36	9.23	28.054	28.024	2.3586	2.3243
0.72	18	54	26.789	26.481	9.178	9.003	9.13	9.02	25.634	25.239	2.3353	2.3276
0.82	18	54	26.434	26.343	9.045	9.001	9.02	8.91	28.165	28.118	2.3323	2.3210
0.92	18	54	26.018	26.011	8.956	8.564	8.96	8.67	40.184	40.019	2.4451	2.4231
1.02	18	54	25.632	25.567	8.767	8.341	8.73	8.43	32.623	32.532	2.5752	2.5421

An excellent correlation was observed (Table 4) observing, mean, variance and standard deviation between the measured and simulated results. Initially it was calculated for measured and simulated frequency from Table 3, using Eq (11)

$$s=\sqrt{\frac{\sum_{i=1}^{n}{\left(x_{i}-\bar{x}\right)}^{2}}{n-1}}$$ (11)

*s* = standard deviation

$\bar{x}$ = mean

*x*_*i*_ = single data

*n* = size of data

**Table 4:** Mean, variance and standard deviation simulated and measured variation of antenna design parameters with substrate thickness at 28GHz.

	*Frequency* (GHz)		*Directivity* (dB)		*Gain* (dBi)		*Return Loss* (dB)		*BW* (GHz)	
	Sim.	Mea.	Sim.	Mea.	Sim.	Mea.	Sim.	Mea.	Sim.	Mea.
**Mean ($\bar{x}$)**	27.083	26.876571	9.1797143	8.9324286	9.1814286	8.94	30.946571	30.783857	2.2447714	2.2387571
**Standard Deviation (*s*)**	1.1680071	1.0453808	0.27553688	0.34982941	0.30808162	0.29614186	8.2001004	8.178104	0.33779699	0.34402982
**Variance (s** ^**2**^ **)**	1.3642407	1.092821	0.075920571	0.12238062	0.094914286	0.0877	67.241646	66.881386	0.11410681	0.11835652

## Results and discussion

### Working principle of hybridization mechanism

In the current study, five different cases based on different modes of switches (PIN Diodes) have been observed for Hybrid (Frequency, Pattern, and Polarization) reconfiguration as shown in Table 5.

**Table 5:** Cases to be observed for analysis of hybrid reconfiguration mechanism.

Case	Switches	Reconfiguration Mechanism
**Case 1**	S1, S2	Frequency Reconfiguration Between P1_28 & P2_38
**Case 2**	S3-S6	Polarization Reconfiguration at P1_28
**Case 3**	S7-S10	Polarization Reconfiguration at P2_38
**Case 4**	S11-S14	Radiation Pattern Reconfiguration at P1_28
**Case 5**	S15-S18	Radiation Pattern Reconfiguration at P2_38

### Frequency reconfiguration

Initially, case 1 was observed for analysis, which displays frequency reconfiguration type between two square patches such as P1_28 (radiates at 28GHz) and P2_38 (radiates at 38GHz). The operating frequency is determined by patch size length (*psl*) and effective permittivity of the of the substrate (*εeff*) and is given by Eqs (12 and 13)

$$opf\;1=\frac{c}{2psl1\sqrt{\varepsilon_{eff}}}$$ (12)

$$opf\;2=\frac{2}{2psl2\sqrt{\varepsilon_{eff}}}$$ (13)

Where c is the speed of light in meter per second square.

**Fig 4:**
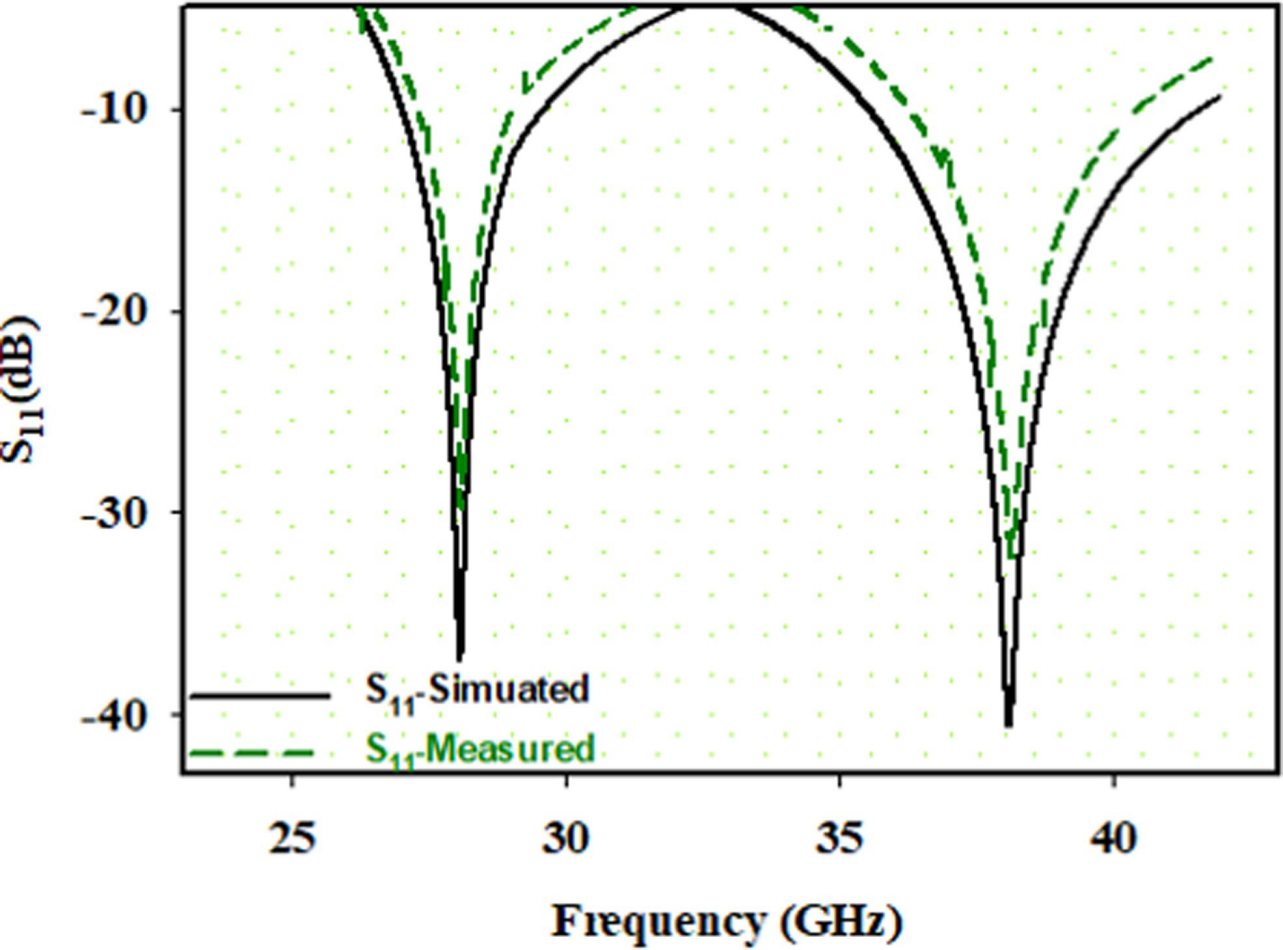
Return loss. S11 result when both S1 and S2 are at ON position.

Both of these patches are connected through S1 and S2. When S1 is at ON state and S2 is at OFF state, P1_28 is active and radiates at 28GHz (*opf1*) resonant frequency (28.02 (simulated) and 28.38 (measured)), showing 15.32% bandwidth (BW). Similarly, when S2 is at ON position and S1 is at OFF, P2_38 is active patch and radiates at 38GHz (*opf2*) (37.96GHz (simulated) and resonant frequency of 38.42GHz (measured)), with 13.65% BW. Furthermore, when both S1 and S2 are at ON state, both the resonant frequencies can be obtained (Fig 4). Additionally, Return Loss (RL) <-10dB for both 28GHz and 38GHz mm-wave frequency band was the finite result of the simulator measured, with a small dissimilarity. These results were due to losses connected with SMK (Sub-Miniature Version K), tangent loss, and improper soldering.

The proposed structure was chosen for 5G cellular smartphones according to the required standard dimensions. The circuit board was manufactured using a photolithography optic-radiation technique that processed the photoresist layer to transfer the mask onto silicon panels. The proposed antenna was designed for the radio frequency and wireless design of the Ansys HFSS (High Frequency Structure Simulator) 19.2 3D electromagnetic field simulator and was tested using a ZVA 40GHz vector network analyzer.

**Fig 5:**
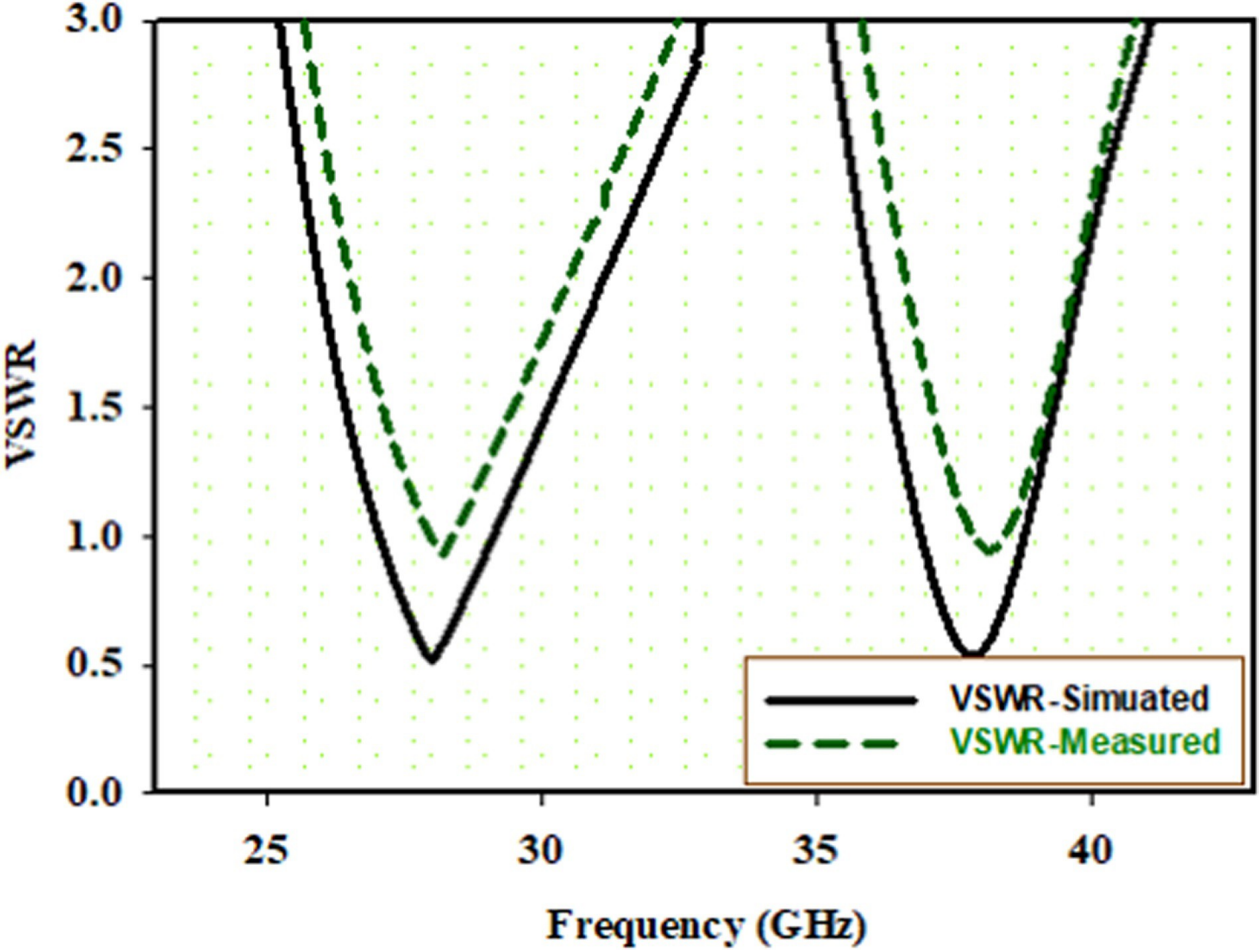
VSWR result when both S1 and S2 are at ON position.

Along with RL, VSWR of both the patches were analyzed, as shown in Fig 5. The surge in VSWR at a frequency close to 28GHZ and between 30GHz in Fig 5. is due to losses that occurs during soldering of SMK connecter in fabrication process or sometimes introduced by diode’s packaging capacitances and inductances which are not included in the simulated results.

### Polarization reconfiguration

In this section different modes of case 2 and case 3 have been discussed. A single-feed microstrip patch can radiate circular polarization (CP) if two orthogonal patches modes TM10 and TM01 are simultaneously excited with equal amplitude ±90° out of phase with the sign determining the sense of rotation. This can be accomplished by perturbing the patches at appropriate locations to the feed. In the proposed design corner Triango-Truncated perturbation is used to get CP, because there is only one parameter to deal with i.e., the depth of truncation *y*. The position of the feed for the perturbation plays an important role in determining the sense of rotation (RHCP/LHCP). For LHCP, the truncations are made on orthogonal corners. With perturbation, the fundamental mode is split into two orthogonal modes TM10 and TM01 with slightly different frequencies *fda* and *fdb* (Eq 14).


$$fda=opf\;1(1-\frac{2\Delta S}{S}),\;fda=opf\;2$$ (14)

Where Δ*S* is the total area of square patches (P1_28 & P2_38) and S is area of square patch i.e. *S = (psl)*^*2*^.

In order to achieve two orthogonal modes with equal amplitude and ± 90° phase shift, corner truncated perturbation has to satisfy the following condition (Eq 15)

$$\frac{\Delta \text{S}}{\text{S}}=\frac{1}{2Q_{0}}$$ (15)

*Q*_*o*_ is unloaded quality factor of two orthogonal modes.

Moreover, good impedance and AR characteristics can be achieved by increasing the substrate thickness. This is because the larger value of y dimensions needs enhanced perturbations to achieve good AR characteristics. However, no shift in resonant frequency with y is observed in AR characteristics. The variation of y dimension is found to have negligible effect on the characteristic.

**Fig 6:**
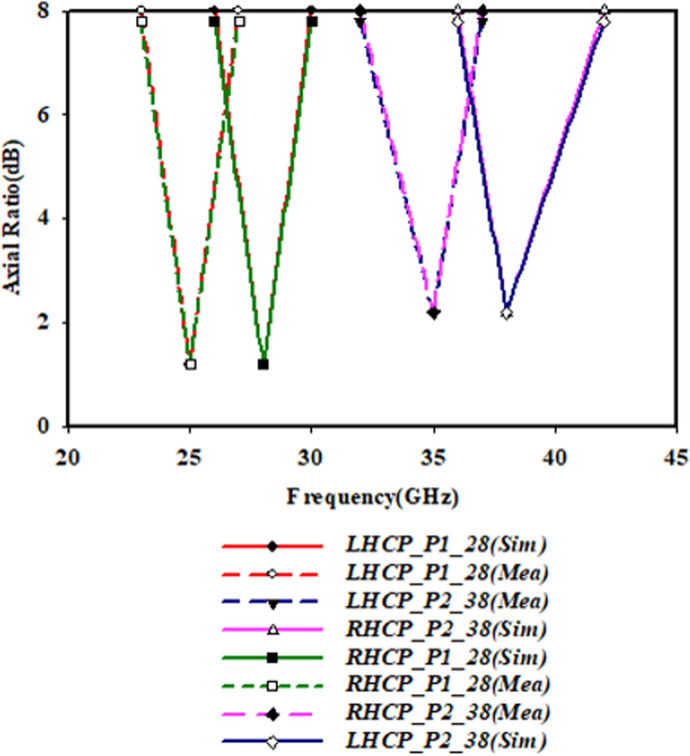
Simulated and measured results of P1_28 & P2_38 patches AR at y = 1.2 mm.

The obtained CP bandwidths referred to 3-dB AR, assessed from simulation results in comparison to measurement results, following the simulated and measured AR of both the patches are shown in Fig 6. Satisfactory agreements are obtained between the measured and simulated results. The discrepancy compared with the simulation may come from several aspects such as the influence of the fabrication error. Due to structure symmetry, the RHCP and LHCP modes have similar results at the same working frequency. This can be observed from Fig 8 where AR in P1_28 is almost the same as in P2_38.

### Radiation pattern reconfiguration

**Fig 7:**
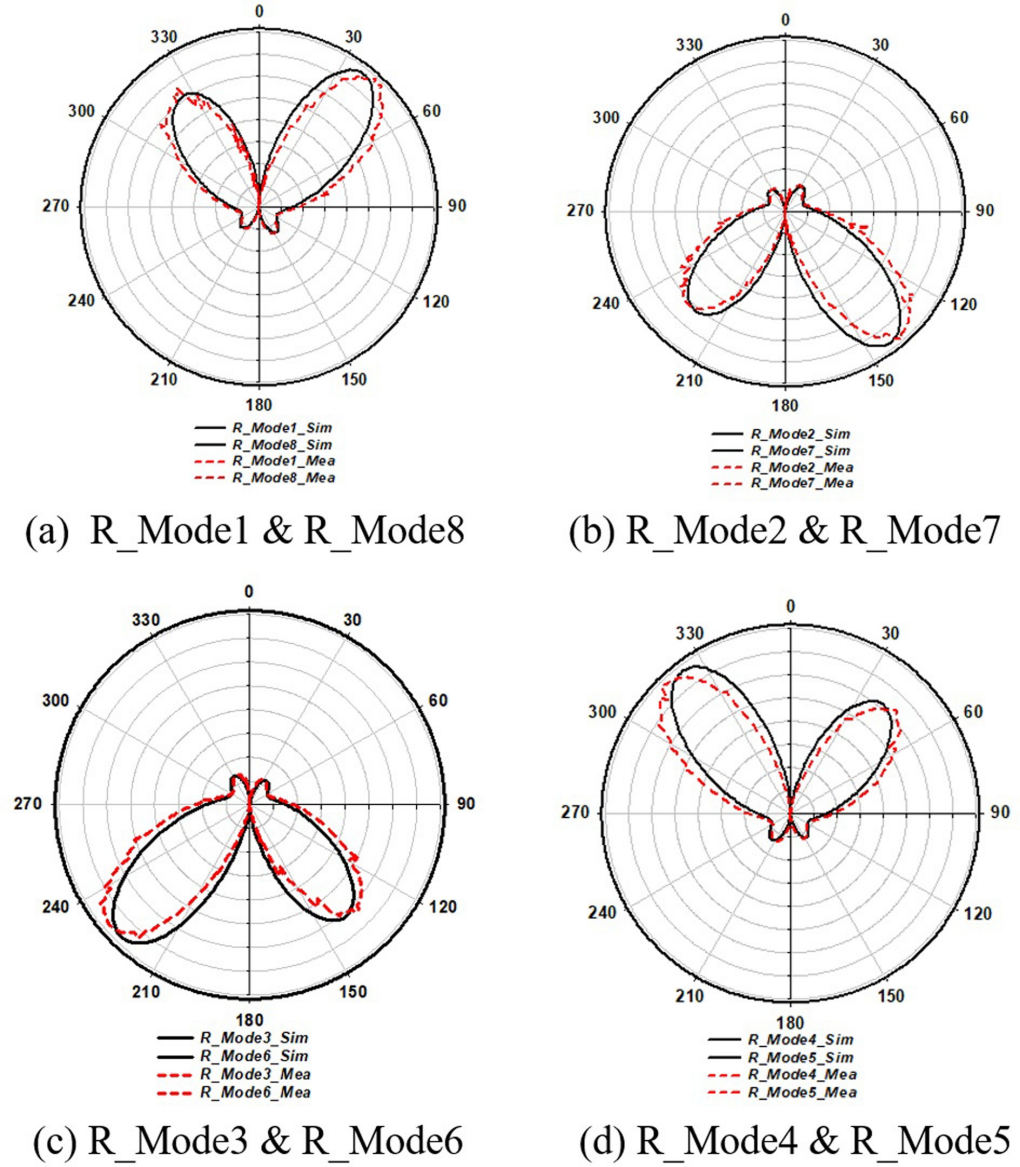
Simulated and measured polarization results showing radiation pattern of both 28GHz and 38GHz on XOY-E-Plane.

In this section we have discussed the pattern reconfiguration of Case 4 and Case 5 at different modes. For pattern steering a parasitic stub were connected with partial ground through PIN Diode switches such as S11-S14 at P1_28 and S15-S18 at P2_38. The measured radiation patterns in XOY-plane P1_28 and P2_38 are illustrated in Fig 7. The radiation patterns in the XOY-plane are almost unchanged for these states, so they are not shown for brevity. It can be seen that the pattern perturbation level is very minor. It is also observed that frequency characteristics are maintained in these states while changing the beam direction when the parasitic stubs elements are switched, which satisfies the principle of pattern reconfigurability and depicting good diversity performance.

However, when the antenna is switched to any of the patch (P1_28 or P2_38), asymmetry is presented in the basic configuration altering the current flow direction on the patch surfaces. Especially, owing to the presence of Triango-Truncated corners, the basic of current is agitated by altering the vector of the surface current in y-direction without changing vector of current directed along x-direction. This makes the resonant mode of current bisection into two orthogonal degenerated modes with 90° phase shift and equal amplitude. Therefore, the instantaneous vector of the surface current on the patch rotates and the radiated fields are circularly polarized. In this reconfiguration process, the beam direction of radiation patterns and resonance frequency are also unchanged.

The purpose of these switches was to yield the change in surface current distribution, which would thus change the characteristics of the radiation pattering. As a result, the radiation and surface current density of the antenna operating at different switching modes was derived as 90° shifts along the XOY-plane (E-plan) *ϕ* = 0°.This direction along the XOY-plane was altered by changing the ON-OFF states of switches. In accordance with the ON-OFF states of the parasitic stubs with symmetrical antenna structures, the single main beam’s direction was symmetrically variant along the *z*-axis in the XOY-plane (Fig 7, from R_Mode1 to R_Mode8). These modes showed narrow HPBW (Half Power Beam width) and a high-quality pattern.

**Fig 7** shows the simulated and measured polarization radiation pattern results of Case 4 & Case 5. It illustrates that at R_Mode1 (Fig 7A) and R_Mode5 (Fig 7D), radiation pattern of the proposed antenna lay between ±0° & ±90° (anti-clock beam rotation) of the P1_28 & P2_38 patches polar plot coordinates. At R_Mode2 (Fig 7B) and R_Mode6 (Fig 7C), the pattern moves in between ±90° & ±180° caused by S12 and S16 at ON states. The pattern then moves to ±180° & ±270° coordinates when S13 and S17 are kept at ON states at R_Mode3 (Fig 7C) and R_Mode7 (Fig 7B). Similarly, At R_Mode4 (Fig 7D) and R_Mode8 (Fig 7A), when S14 and S18 is at ON position, the radiation pattern shifts in between ±270° & ±360°, resulting in a radiation pattern mechanism, respectively. Additionally, Fig 7 represent the changing positions of the pattern through switches (anti-clockwise) cause the surface current density to vary, due to the connecting parasitic stubs. Depending on the number of stubs, we can increase/decrease the steering angle (90° shift is used in this study).

### Hybrid reconfigurable structure for harvesting

RF energy is inversely proportional to distance and therefore drops as the distance from a source is increased. Harvested power from RF energy sources is lower than 0.1mW/cm2. Electromagnetic energy harvesting system is shown in Fig 1. A hybrid reconfigurable antenna can harvest RF energy from 28GHz up to 38GHz. The received RF energy may be combined after transformed to DC power. The RF energy harvesting system consists of antennas, matching and feed networks, rectifying circuit, and a rechargeable battery. The harvesting energy system operates as a dual mode RF harvesting system. The harvesting unit can be part of a medical, IOT, computer, and smartphone. The DC bias voltages are supplied by the receiving system. The energy coupled to the transmitting built in test may be harvested and used to charge a battery. We can calculate the energy harvesting link budget by using Eqs (16–26), [31], if the antennas are matched and there are no losses in the medium. However, if the antennas are not matched and the RF energy propagates in a loss media, we can calculate the energy harvesting link budget. Wireless smart phone can transmit up to a maximum about 200mW (which is 23dBm). Free Space Loss *L*_*p*_ represents propagation loss in free space. Losses due to attenuation in atmosphere, $L_{a}=e^{-\alpha_{c}r}$, should be accounted for in the transmission equation

$$P_{r}=P_{t}G_{t}G_{r}{\left(\frac{\lambda }{4\pi R}\right)}^{2}$$ (16)

$$P_{r}=P_{t}G_{t}G_{r}{\left(\frac{\lambda }{4\pi R}\right)}^{2}\left(1-{\left|\Gamma_{t}\right|}^{2}\right)\left(1-{\left|\Gamma_{r}\right|}^{2}\right){\left|a_{t}a_{r}{}^{*}\right|}^{2}e^{-\alpha_{c}r},\alpha_{c}=\text{attenuation}\;\text{constant}$$ (17)

$$L_{p}={\left(\frac{4\pi R}{\lambda }\right)}^{2}$$ (18)

The received power is given as,

$$P_{r}=\frac{P_{t}G_{t}G_{r}}{L_{p}}$$ (19)

Polarization or mismatch losses,

$$L_{pol}={\left|a_{t}a_{r}\right|}^{2}$$ (20)

Receiving antenna losses,

$$L_{r}=(1-{\left|\Gamma_{r}\right|}^{2})$$ (21)

Transmitting antenna losses,

$$L_{ta}=(1-{\left|\Gamma_{t}\right|}^{2}),\;\Gamma_{r}\;\text{is}\;\text{reflection}\;\text{coefficient}\;\text{of}\;\text{receiving}\;\text{antenna}$$ (22)

$$P_{r}=\frac{P_{t}G_{t}G_{r}}{L_{p}L_{a}L_{ta}L_{ra}L_{pol}L_{other}L_{r}}$$ (23)

Γ_*t*_ is reflection coefficient of transmitting antenna

*P*_*t*_ = transmitting power

*L*_*t*_ = Loss betweenpower sourceand antenna

EIRP = effective isotropic radiated power = *P*_*t*_*G*_*t*_

Where,

$$P_{r}=\frac{EIRP\times G_{r}}{L_{p}L_{a}L_{ta}L_{ra}L_{pol}L_{other}L_{r}}$$ (24)

$$P_{r}=\frac{P_{out}G_{t}G_{r}}{L_{t}L_{p}L_{a}L_{ta}L_{ra}L_{pol}L_{other}L_{r}}$$ (25)

The received power in dBm may be calculated by

$$P_{r}=EIRP-L_{p}-L_{a}-L_{ta}-L_{ra}-L_{pol}-L_{other}-L_{r}-G_{r}$$ (26)

Based on the above Eqs (16–26) the following power has been harvested (Table 6)

**Table 6:** Power harvested at different radiation modes.

Freq.	Active patch	Active radiation pattern	Peak Efficiency (%)	Power harvested at peak efficiency (mW)
28GHz	P1_28	R_Mode1	78.3	61.2
		R_Mode2	76.7	62.4
		R_Mode3	77.5	61.1
		R_Mode4	78.1	61.8
38GHz	P1_38	R_Mode5	72.6	56.4
		R_Mode6	71	56
		R_Mode7	72	56
		R_Mode8	72.4	56.1

**Fig 8:**
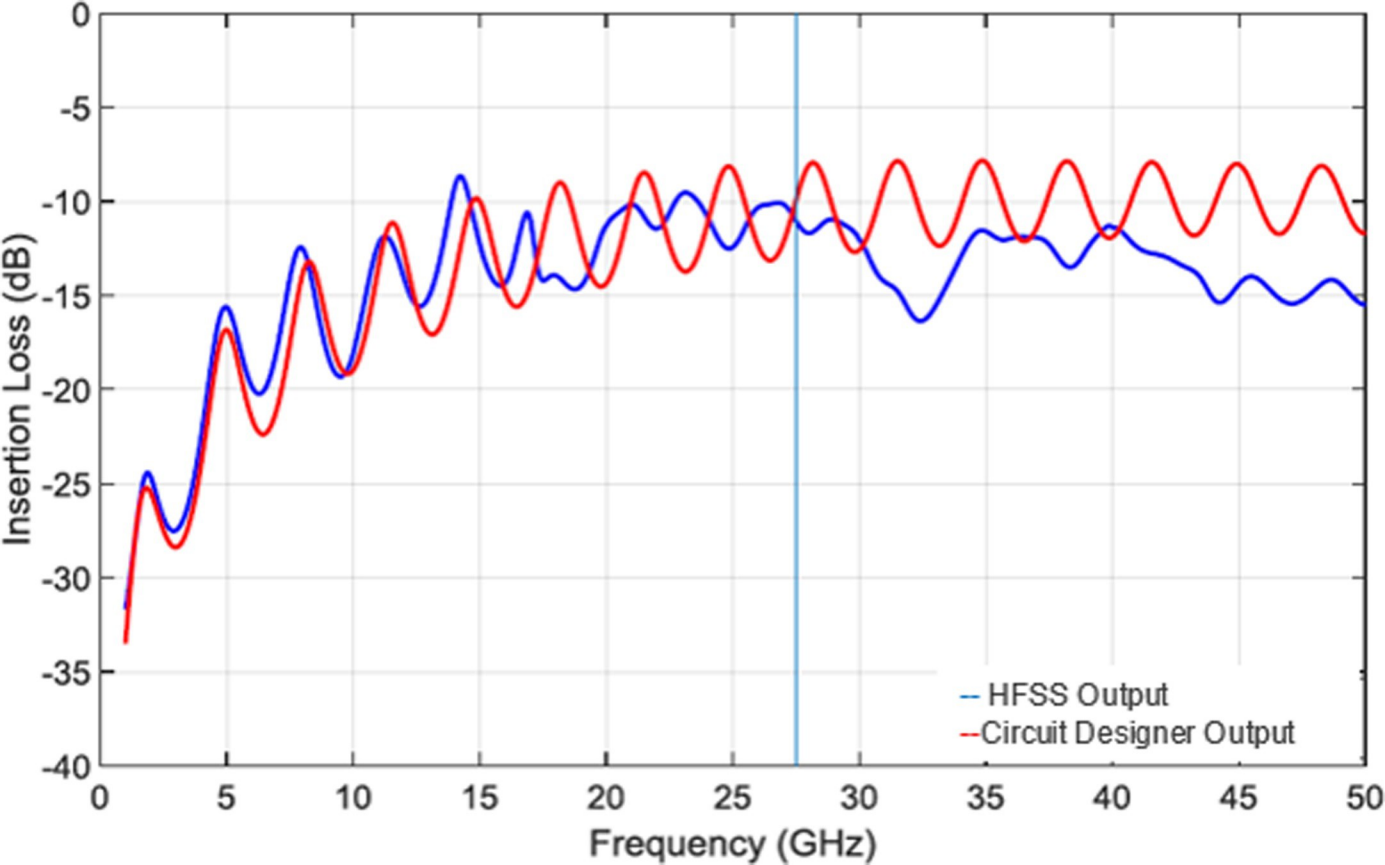
Comparison of Circuit Design and HFSS Design for the same structure when P1_28 GHz is active.

Initially we set our simulation from 1 to 50GHz with step 0.01GHz with 4901 points. The efficiency of the antenna is measured through the return loss, or S11. The antenna will radiate within the frequency range where *S*11<−10*dB*. Then stubs (used for radiation pattern) of length *L = 2*.*4mm* and width W = 0.8mm on the ground of each patch was analyzed and compared with RLC equivalent circuit analyzer. For this purpose, insertion loss was observed to validate the matching impedance at *Z = 50ohm* (as shown in Fig 8).

Table 5 shows that using different radiation pattern modes, a total of 1W or above power can be achieved and stored with help of hybrid reconfigurable antenna at 28GHz and 38GHz resonant frequency. The harvested voltage from the three antennas is *V*_*total*_ as written in Eq (27). The total DC energy is *P*_*total*_ as written in Eq (28). The actual harvested energy is *P* as written in Eq (29).


$$\eta =\frac{DCoutputpower}{ACinputpower}=\frac{{\left(\frac{2I_{m}}{x}\right)}^{2}R}{{\left(\frac{I_{m}}{2}\right)}^{2}(R+rf)}0.812$$ (27)

*V*_*total*_ = voltages at all modes = *V*_1_ + ……+ *V*_8_ ≈ *V*_*nth*_ (for nth order)

$$P_{total}=\frac{V_{total}^{2}}{R}=I_{m}^{2}R$$ (28)

$$P=0.182\;P_{total}=0.182\frac{V_{total}^{2}}{R}=0.182I_{m}^{2}R$$ (29)

Our investigation results were compared with the previously published literature, as shown in Table 7. The major attributes of the proposed model are as follows:

- The proposed model is design for Frequency, pattern and polarization reconfiguration, independently controlled in a single antenna, showing structural novelty.
- A single antenna with different resonant frequency i.e. 28/38GHz used as Rectenna for RF energy Harvesting.

**Table 7:** Comparison table of published literature.

Ref. No	Freq.	Reconfiguration mechanism	Gen.	Application
[32]	4.64GHz	Polarization diversity (LHCP or RHCP)	4G	WLANs, satellite links, and space robots
[33]	4.44GHz and 4.49GHz	Polarization diversity (LHCP or RHCP)	4G	Unlicensed and licensed WiMax (IEEE 802.16a), future planetary missions, and satellite links
[34]	2.45GHz	Polarization diversity (LHCP or RHCP)	4G	Not available
[35]	Covers five different bands (from 2GHz up to 7GHz)	Frequency diversity	4G	Cognitive radio systems
[36]	2.5 2.55GHz and 3.4 3.7GHz WiMax bands	Frequency diversity	4G	Covers 2.5 2.55GHz and 3.4 to 3.7GHz WiMax bands
[37]	5.15 5.35GHz and 5.5GHz	Frequency diversity and polarization diversity (LHCP or RHCP)	4G	Can function as a rectenna for wireless battery charging at 5.5GHz and data telemetry in the 5.15–5.35GHz WLAN band
[38]	2.415GHz and 2.650GHz	Frequency diversity and polarization diversity (LP, LHCP, or RHCP)	4G	WLAN/digital, multimedia broadcasting applications
This Work	28GHz and 38GHz	Frequency, Radiation Pattern & Polarization diversity (LP, LHCP, or RHCP)	5G	mm-Wave 5G mobile communication, RF Energy Harvesting

## Conclusion

A hybrid reconfigurable structure of a single feed microstrip patch antenna for future 5G mobile communication applications is presented. The hybridization mechanism is based on frequency, radiation pattern and polarization reconfiguration to capture large amount of energy harvesting. 18 switches are deployed as ON/OFF states, such as 2 for frequency reconfiguration between 28 and 38GHz, 8 for radiation pattern reconfiguration showing a drift of ±90° anticlockwise direction and 8 for polarization such as LP, RHCP and LHCP. The 5G antenna is worked in the 28GHz mm-wave band (27.94–28.83GHz) and the 38GHz mm-wave band (37.97–38.96GHz). The two bands obtained cover millimeter-wave frequency bands for future mobile cellular devices and rectenna as RF energy harvesting. The harvesting energy system operates as a Dual Mode Energy harvesting system i.e. 28GHz and 38GHz, producing a total of 1W power.
